# TGF-β1 relieves burn injury induced pain by alleviating inflammation in mouse

**DOI:** 10.1371/journal.pone.0342029

**Published:** 2026-02-05

**Authors:** Shengfeng Gao, Mengting Wang, Lu Wang, Yasu Jiang, Peng Chen, Li Guo, Lili Ni, Fan Fei, Zhenhua Gong

**Affiliations:** Department of Burn and Plastic Surgery, Nantong First People’s Hospital, Nantong, Jiangsu, China; National University of Medical Sciences, PAKISTAN

## Abstract

Burn injuries are severe traumas characterized by tissue damage and inflammation, and pain is the common symptom of burn patients. Transforming growth factor beta 1 (TGF-β1) is a multifunctional cytokine involved in organ development, immune response, tumor biology and injury repair. This study investigates the effects of TGF-β1 on the burn injury induced pain and explore the underlying mechanisms. A mouse model of second degree burn injury was established, and spinal intrathecal injection with lentivirus was used to knockdown or overexpress TGF-β1. The assessment of mechanical allodynia and thermal hyperalgesia was adapted to evaluate the impact of TGF-β1 on the burn injury induced pain. The expressions of inflammatory factors were measured by using RT-qPCR, while immunofluorescence staining was employed to detect the effects of TGF-β1 on macrophage infiltration in the burned plantar skin. Western blot was used to analyze the effects of TGF-β1 on microglia, astrocytes and signal pathway. RT-qPCR results demonstrated that lentiviruses injection could knockdown or overexpress TGF-β1 in ipsilateral spinal cord, and reduce pro-inflammatory factors (IL-1β, IL-6 and TNF-α) expression and promote anti-inflammatory factor (IL-10) expression. The behavioral assessments revealed that TGF-β1 overexpression alleviated mechanical allodynia and thermal hyperalgesia. Immunofluorescence staining and Western blot revealed that TGF-β1 reduced the macrophage infiltration in the plantar skin, inhibited expressions of marker proteins of microglia and astrocyte, and promoted the phosphorylation of smad2. These findings suggested that TGF-β1 mitigated burn injury induced pain by attenuating inflammatory response via TGF-β1/smad2 pathway. This study provides an experimental and theoretical basis supporting the potential use of TGF-β1 as an anti-inflammatory and analgesic for burn injury.

## Introduction

Burn is a severe trauma in clinic and tissue damage induced by heat source, which can lead to severe infection and complications resulting in weakening of patients’ physiological functions [1,2]. These injuries are associated with high disability rates, low cure rates, and elevated mortality, making them a significant challenge in clinical practice [3]. Up to now, it is still a major challenge of the clinical treatment of burn patients with difficult wound healing, easy infection, severe persistent pain and hypertrophic scar [3,4]. Pain is one of the most common complaints of burn patients [5]. Existing studies believe that the early pain of burn patients is mainly caused by the inflammatory response induced by acute burn stimulation or tissue injury, while the later stage of burn wound healing will appear temporary or continuous burning like neuropathic pain [6,7]. Therefore, an effective analgesic regimen is essential for burn care, as it can accelerate wound healing and patient rehabilitation.

The common drugs for burn pain treatment include nonsteroidal anti-inflammatory drugs (NSAIDs), opioid analgesics and benzodiazepine anxiolytics [8,9]. Although opioids analgesics dominate burn pain management, they are frequently accompanied by severe side effects [10,11]. What is more serious is that long-term using of opioids analgesics will lead to obvious drug resistance, resulting in patients needing larger doses of drugs to maintain the analgesic effect, and finally further aggravate the occurrence of other side effects [12,13]. In clinic, opioids combined with anxiolytics, antidepressants and NMDA receptor inhibitors are mainly used to relieve burn induced pain, but there is still a lack of efficient analgesia for burn induced pain [14,15]. In recent years, a large amount of research has been devoted to the discovery of analgesic therapy with high efficiency and low side effects for the clinical treatment of burn induced pain.

Transforming growth factor beta (TGF-β) is a multifunctional cytokine, with its superfamily members widely distributed in physiological tissues [16,17]. The diverse functions of members of the TGF-β family include cell differentiation, proliferation, migration, angiogenesis, immune response, and extracellular matrix (ECM) deposition, which are crucial for maintaining tissue homeostasis [18–20]. Dysregulation of TGF-β expression can lead to various diseases, including fibrosis, autoimmune disorders, inflammatory responses, and cancer [21,22]. The TGF-β family consists of three isoforms (TGF-β1, TGF-β2 and TGF-β3), which share nearly 70% homology and similar biological activities [23]. TGF-β1 can regulate most of its biological activities [24]. Although there is significant diversity among these isoforms, they also share many cell surface receptors and downstream signaling pathways that regulate cellular biological activities as members of the TGF-β family [17,24]. TGF-β family member ligands can recognize and bind to TGFβR1 and TGFβR2 [25]. TGF-β1 is a multifunctional growth factor that plays important roles in organ development, inflammatory reaction, tumorigenesis and development, and tissue repair [26,27]. Numerous studies have shown that the dysregulation of the TGF-β1/Smad signaling pathway is an important pathogenic key point in the occurrence and development of fibrosis and inflammation [28,29]. Therefore, TGF-β1 and it’s signaling pathway have become important targets for anti-fibrotic and anti-inflammatory drug research. Neuropathic pain is always considered a neuroimmune disorder caused by a complex crosstalk between neurons, activated glia, and immune cells in nervous system. And, growth factors, inflammatory mediators, and cytokines are key players in the pathological plasticity underlying neuropathic pain. So, we proposed that TGF-β signaling plays an important role in tissue repair and burn injury pain. In this study, we studied the effects of TGF-β1 on the burn injury induced pain and seek the mechanism. Our findings provided a novel perspective of TGF-β1 in the management of pain.

## Methods

### Animals

In the study, the adult male C57BL/6 mice, aged 10–12 weeks and weighing 22 ± 2 g were used. All animals were obtained from the Laboratory Animal Center of Nantong University in this study. The experimental mice were housed in a constant temperature (22 ± 1 °C) and humidity environment with a 12 hour light/dark cycle. Five mice were housed in a cage with free access to food and water throughout the experimental period.

### Animal models

Due to the cause of burn is complex and varied, it is generally classified according to the degree of damage of the burn site to quickly determine the treatment plan in clinic. Second-degree burns, which involve partial-thickness injury to the superficial dermis, are among the most common types of burn injuries [30]. This second degree burn injury can induce sharp pain, and the wound needs to be bandaged and nursed. To investigate the effects of TGF-β1 on burn-induced pain, we utilized a mouse model of second-degree burn injury.

The establishment of a mouse burn injury model was based on previous reported methods with partially modification [31]. In brief, the mice were treated with a compound anesthetic (10 mg/kg xylazine, 95 mg/kg ketamine, 0.7 mg/kg acepromazine) and maintained. Then, the right hind paw of the mice was placed on a 65 °C metal plate and kept for 15 seconds. A 50 g weight was placed on the right hind paw to maintain consistent pressure. The sham group mice were subjected to the same treatment on a metal plate at 23 °C. After burn was completed, the mice were taken back into home cage for recovery.

Under the guide for the Care and Use of Laboratory Animals, and experimental mice protocols were approved by the Institutional Animal Care and Use Committee of Nantong University (approval No. IACUC20231220−019). According to the Animal Research: Reporting of *In Vivo* Experiments (ARRIVE) guideline, we conducted animal experiments and reported.

### Behavioral tests

In the study, behavioral tests included evaluations of mechanical allodynia and thermal hyperalgesia. All mice for behavioral tests should be fully familiarized with the testing environment at least two days before baseline (BL) testing. The investigator conducting the behavioral tests was blinded to group assignments.

### Assessment of mechanical allodynia

According to previous reports, the mechanical allodynia was measured by a Von Frey Hairs (Ugo basile, Italy) [32]. In brief, the mice were placed in the perforated metal grid platform and allowed for habituating in the environment 1 h before testing. A series of monofilament was applied perpendicularly to the plantar surface of the hind paw. The paw withdrawal thresholds (PWTs) of mice were determined as the maximum force (g) to induce paw withdrawal response. During performance, the BL threshold was measured 5 times to calculate the average value. However, PWTs were measured 3 times at other time points to calculate the average value.

### Assessment of thermal hyperalgesia

The thermal hyperalgesia was measured by Hargreaves Test instrument (ZL-024E, Anhui Yaokun Biotechnology Co., Ltd, China). Briefly, the mice were placed in a transparent plexiglass box with a glass bottom that can transmit infrared radiation. Before testing, mice were familiar the environment for at least 30 m. An infrared heat source from the bottom of the glass was applied to directly stimulate the mid-plantar skin of hind paws of mice, and withdrawal time was recorded. The paw withdrawal latencies (PWLs) were determined as the time that a mouse withdrew its paw. 20 s as cut-off time was used to prevent skin damage.

### Hematoxylin-Eosin (H&E) staining

24 hours after burn injury, the mice were euthanized via CO_2_ inhalation, and the plantar skin tissue of the right hind paw was immediately removed and fixed in 4% paraformaldehyde (PFA) overnight. The plantar skin after fixed was embedded in paraffin, and then cut into 4 μm thick sections. The sections were dewaxed with xylene and hydrated with ethanol gradient before H&E staining. After staining, ethanol with different concentration was used for gradient dehydration, and neutral resin was used for sealing. Finally, observe and take photos through the microscope. Histological damage was quantitatively assessed using a blinded scoring system, as previously described in the literature [33,34].

### Spinal intrathecal injection

The recombinant lentiviral vectors were constructed to knockdown TGF-β1 (Lv-sh) or overexpress TGF-β1 (Lv-TGF). The empty lentiviral vector was used as negative control (Lv-sh-NC and Lv-TGF-NC). The used lentivirus was provided by Vigen Biotechnology (Zhenjiang) Co., Ltd. The average titer of lentivirus was 2 × 10^8^ plaque forming unit (PFU)/ml. based on previously described [35], the glass electrode needle connected to the microinjector was inserted obliquely through the L4-L5 intervertebral space where bony parts are absent, and 10 μl lentivirus each mouse was injected with a Micro4 microinjection pump.

### Immunofluorescence staining

After anesthesia with intraperitoneal injection of compound anesthetics, the chest of mice was opened, and then normal saline was used to flush the blood through the left ventricle. The 4% PFA replaced normal saline was further used to complete pre-fixation. Following, the L4-L5 spinal cord tissues and right plantar skin were quickly collected and post-fixed in 4% PFA overnight. The fixed L4-L5 spinal cord and plantar skin tissue was embedded in paraffin, and then cut into 4 μm thick sections. The tissues were respectively reacted with primary antibodies at 4 °C overnight, and then reacted with corresponding second antibodies for 2 h at 37 °C. Additionally, these tissues were also stained with DAPI at 37 °C for 10 m. ZEISS Axio Scope.A1 fluorescence microscope (Oberkochen, Germany) was adapted to observe fluorescence signal. The used primary and second antibodies were recorded in the Table 1.

**Table 1:** The used antibodies.

Antibody	Sources	Clone number	Catalogue number
β-actin	Sigma-Aldrich	AC-15	A3854
Iba1	Abcam	EPR16588	ab178846
GFAP	Abcam		ab7260
p-smad2	Cell Signaling	E8F3R	18338
smad2	Cell Signaling	D43B4	5339
TGF-β1	Abcam	EPR21143	ab215715
Anti-mouse IgG HRP	Sigma-Aldrich		A0168
Anti-rabbit IgG HRP	Sigma-Aldrich		AP510P
Anti-rabbit IgG 488	Abcam		ab150077

### RT-qPCR

The L4-L5 ipsilateral spinal cord tissues and the plantar skin tissues 5 d after burn injury induced pain were collected, and homogenized in TRIzol solution (ThermoFisher scientific, Waltham, MA) using a homogenizer to extract total RNA. Then the reverse transcription kit (QIAGEN, Hilden, Germany) was used to synthesize the first-strand cDNA from total RNA. The sequences of used primers were exhibited in the Table 2. Quantitative PCR was performed using a QuantStudio 3 (ThermoFisher Scientific, Waltham, MA) and SYBR Green Mix (Yeasen Biotechnology, Shanghai, China). GAPDH was used as a quantitative normalization. Each sample was measured in triplicate and then the 2^−ΔΔCT^ method was adapted for the analysis of the relative transcription data.

**Table 2:** The sequences of primers.

Gene (GenBank accession number)	Primer	Sequence (5’-3’)
GAPDH (NM_001289726)	Forward	TGGAGAAACCTGCCAAGTATG
	Reverse	ATGTAGGCCATGAGGTCCAC
IL-1β (NM_008361)	Forward	CAGGCAGGCAGTATCACTCA
	Reverse	GCCCAAGGCCACAGGTAT
IL-6 (NM_031168)	Forward	GGAGCCCACCAAGAACGATA
	Reverse	CAGGTCTGTTGGGAGTGGTA
IL-10 (NM_010548)	Forward	AGCCGGGAAGACAATAACTG
	Reverse	TCACTCTTCACCTGCTCCAC
TNF-α (NM_013693)	Forward	TGCCTATGTCTCAGCCTCTTC
	Reverse	CTCCTCCACTTGGTGGTTTG
TGF-β1 (NM_011577)	Forward	AGAGACGTGGGGACTTCTTG
	Reverse	GCTTTGGGGTGAAGTCTTCG
CD86 (NM_019388)	Forward	CAGCACGGACTTGAACAACC
	Reverse	CTCCACGGAAACAGCATCTGA
Arg1 (NM_007482)	Forward	CATATCTGCCAAAGACATCGTG
	Reverse	GACATCAAAGCTCAGGTGAATC

### Western blot

The L4-L5 ipsilateral spinal cord tissues were harvested, and dissociated in RIPA lysis buffer (MERCK, Darmstadt, Germany). The protein was quantified by BCA Protein Assay Kit (Yeasen Biotechnology, Shanghai, China), and protein was transferred to PVDF membrane after SDS-PAGE electrophoresis separation. The membrane was blocked with 5% non-fat milk for 2 h at room temperature, and respectively incubated with primary antibodies overnight at 4 °C. Then, the membrane after washing was incubated with HRP-conjugated second antibodies for 2 h at 37 °C. β-actin was used as a quantitative normalization. The used primary and second antibodies were recorded in Table 1. ImageJ software (NIH, Bethesda, MD) was applied to quantify corresponding protein band.

### Statistical analysis

All data were stated as means ± Standard Error of Mean (SEM). Data were analyzed using GraphPad Prism software (version 8) (GraphPad, La Jolla, CA). One-way ANOVA with subsequent Turkey’s tests was applied to analyze statistical differences between groups. Two-way ANOVA with Bonferroni post-hoc test was applied for multiple comparisons. *p* < 0.05 was considered statistically significant difference.

## Results

### Burn injury model and induced pain

**Fig 1:**
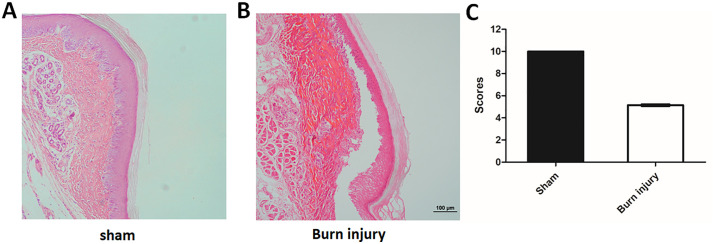
H&E staining of plantar skin tissue of right hind paw in mice. (A) the sham group, (B) the burn injury group. Scale bar = 100 μm. (C) quantitative analysis of histological damage using blinded scoring. n = 5. H&E, Haematoxylin and Eosin.

To investigate the effects of burn injury on tissue morphology and pain behavior, we established a mouse model of second-degree burn injury. In the sham group, obvious unusual was not observed in the gross morphology of the hind plantar skin of mice. Histological examination of H&E staining also revealed that the tissue structure of the hind plantar skin of mice in the sham group was normal and intact. However, in the burn injury group, evident redness and swelling could be observed on the right hind paw of mice, indicating the existence of local inflammatory response. Based on H&E staining, the tissue structure of the hind plantar skin of mice was severely damaged, and the characteristics of second degree burn in the burn injury group were observed (Fig 1A-B). The epidermis was missing, and the dermis was damaged in burn injured mice, which were the typical display of the mouse model of second degree burn injury. The epidermis was absent, and the dermis was extensively damaged, confirming the successful establishment of the second-degree burn injury model. Additionally, histological scoring further demonstrated that the tissue integrity in the burn injury group was significantly compromised compared to the sham control (Fig 1C).

**Fig 2:**
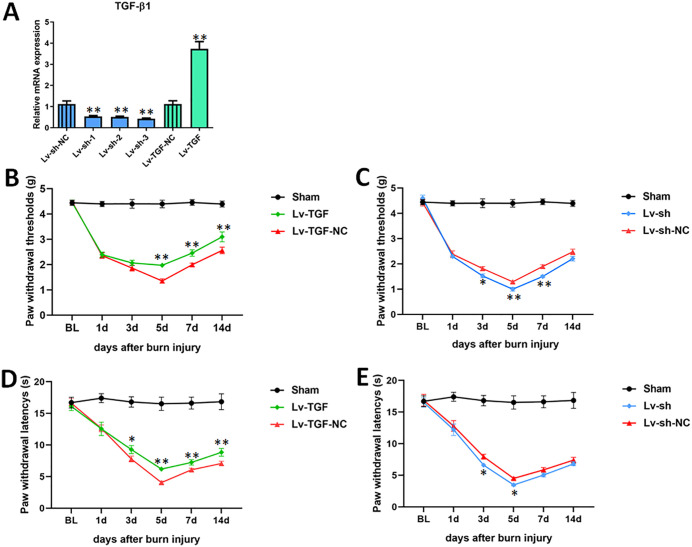
Effects of TGF-β1 on burn injury induced pain. After mice models of second degree burn injury were established, the lentiviruses were intrathecally injected into the L4-L5 foramina. (A) the mRNA expressions of TGF-β1 were detected by RT-qPCR in L4-L5 ipsilateral spinal cord tissues after injected with overexpression lentivirus (Lv-TGF) or knockdown lentivirus (Lv-sh) for 3 days. (B) and (C) the paw withdrawal thresholds (PWTs) (g) of mice were measured by using a Von Frey Hairs for assessment of mechanical allodynia. (B) TGF-β1 overexpression and (C) knockdown of TGF-β1. (D) and (E) the paw withdrawal latencys (PWLs) (s) of mice were measured by using Hargreaves Test for assessment of thermal hyperalgesia. (D) TGF-β1 overexpression and (E) knockdown of TGF-β1. Versus Lv-sh-NC or Lv-TGF-NC, *p < 0.05, and **p < 0.01. n = 5. TGF-β1, transforming growth factor-beta 1. RT-qPCR, real-time quantitative PCR. shRNA, small hairpin RNA. Lv-sh, shRNA lentivirus. Lv-sh-NC, shRNA lentivirus negative control. Lv-TGF, TGF overexpression lentivirus. Lv-TGF-NC, TGF overexpression lentivirus negative control. BL, baseline.

We measured the burn injury induced pain by assessment of mechanical allodynia and thermal hyperalgesia at 1, 3, 5, 7 and 14 days after burn injured. The results showed that the PWTs of mice in sham group under mechanical stimulation and PWLs under photothermal stimulation were no significant differences, compared to baseline (BL) (*p* > 0.05) (Fig 2). As anticipated, the PWTs and PWLs of mice in burn injury group were all obvious changes.

### TGF-β1 relieved burn injury induced pain

After mice models of second degree burn injury were established, the lentiviruses were intrathecally injected into the L4-L5 foramina. RT-qPCR was applied to detect the mRNA expressions of TGF-β1 in L4-L5 ipsilateral spinal cord tissues after injected with overexpression lentivirus (Lv-TGF) or knockdown lentivirus (Lv-sh) for 3 days. The results of RT-qPCR revealed that the mRNA expressions of TGF-β1 were remarkably increased after injected with Lv-TGF (*p* < 0.01), compared to negative control (Lv-TGF-NC) (Fig 2A). And the mRNA expressions of TGF-β1 were all notably decreased after injected with knockdown lentivirus (Lv-sh-1, Lv-sh-2 and Lv-sh-3). The down regulation of mRNA expression of TGF-β1 was most significant in Lv-sh-3 group. Therefore, in the next experiments, the Lv-sh-3 was used.

The lentiviruses could be applied to overexpress or knockdown TGF-β1. Next, we investigated the effects of TGF-β1 on the burn injury induced pain. Von Frey Hairs was adapted to measure the PWTs for assessment of mechanical allodynia, and Hargreaves Test was adapted to measure the PWLs for assessment of thermal hyperalgesia. The Von Frey Test results displayed that the PWTs were evidently raised in Lv-TGF group, compared to Lv-TGF-NC (*p* < 0.01), at 5, 7 and 14 d after burn injury induced pain (Fig 2B). Conversely, the PWTs were obviously descended in TGF-β1 knockdown group at 3, 5, and 7 d after burn injury induced pain (Fig 2C). The results of assessment of mechanical allodynia were similar to that of Von Frey Test (Fig 2D and E). The results of Von Frey Test and Hargreaves Test both indicated that TGF-β1 could relieve the burn injury induced pain.

### Effects of TGF-β1 on inflammatory factors in pain

**Fig 3:**
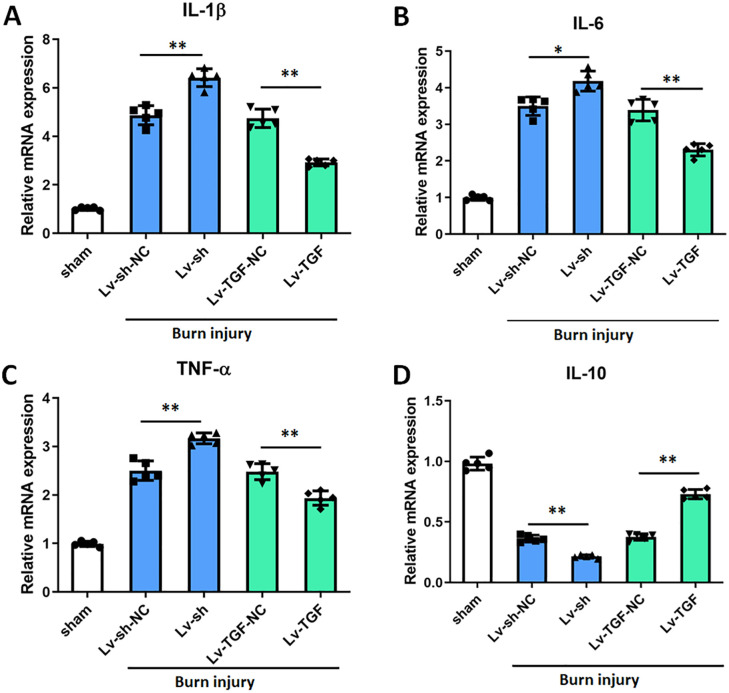
Effects of TGF-β1 on inflammatory factors. RT-qPCR was applied to detect the effects of TGF-β1 on mRNA expressions of inflammatory factors in L4-L5 ipsilateral spinal cord tissues. (A-C) the mRNA expressions of pro-inflammatory factors (IL-1β, IL-6 and TNF-α). (D) the mRNA expressions of anti-inflammatory factors (IL-10). *p < 0.05, and **p < 0.01. n = 5. TGF-β1, transforming growth factor-beta 1. RT-qPCR, real-time quantitative PCR. shRNA, small hairpin RNA. Lv-sh, shRNA lentivirus. Lv-sh-NC, shRNA lentivirus negative control. Lv-TGF, TGF overexpression lentivirus. Lv-TGF-NC, TGF overexpression lentivirus negative control.

Pain is a consequence of inflammation triggered by tissue injury, and inflammatory factors are implicated in both the initiation and persistence of pathologic pain [36]. To elucidate the role of TGF-β1 in modulating inflammation, we assessed the effects of TGF-β1 on mRNA expressions of inflammatory factors (IL-1β, IL-6, TNF-α and IL-10) by RT-qPCR in L4-L5 ipsilateral spinal cord tissues. The results of RT-qPCR showed that the mRNA expressions of pro-inflammatory factors (IL-1β, IL-6 and TNF-α) were all dramatically downregulated, however the mRNA expression of anti-inflammatory factor (IL-10) was significantly upregulated after injected with (Lv-TGF) (Fig 3). On the contrary, the IL-1β, IL-6 and TNF-α mRNA expressions were all evidently raised, and IL-10 was prominently declined after knockdown of TGF-β1. The above results revealed that TGF-β1 could restrain pro-inflammatory factors expressions and promote anti-inflammatory factors expressions in burn injury model.

### TGF-β1 reduced the infiltrated macrophages

**Fig 4:**
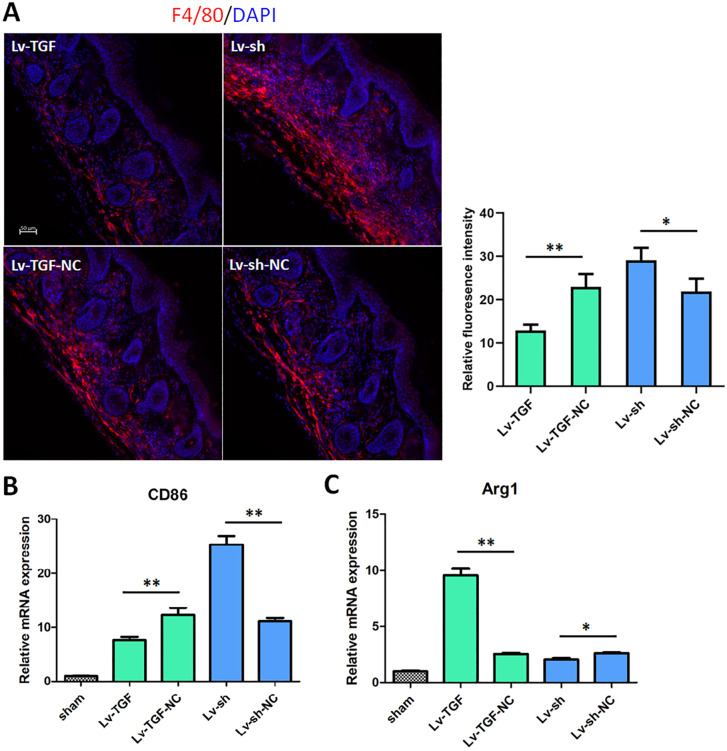
TGF-β1 reduced the infiltrated macrophages. (A) At 5 d after burn injury induced pain, the plantar skin tissues were immunofluorescence stained with anti-F4/80 antibody for detecting macrophages. The red was the positive signal of F4/80. Bar = 50 μm. (B) and (C) the mRNA expressions of M1 marker (CD86) and M2 marker (Arg1) were measured by RT-qPCR in plantar skin tissues. *p < 0.05, and **p < 0.01. n = 5. TGF-β1, transforming growth factor-beta 1. RT-qPCR, real-time quantitative PCR. CD86, cluster of differentiation 86. Arg1, Arginase 1. shRNA, small hairpin RNA. Lv-sh, shRNA lentivirus. Lv-sh-NC, shRNA lentivirus negative control. Lv-TGF, TGF overexpression lentivirus. Lv-TGF-NC, TGF overexpression lentivirus negative control.

To examine the role of TGF-β1 in macrophage infiltration and polarization, plantar skin tissues were collected 5 days post-burn injury and stained with an anti-F4/80 antibody. Based on the immunofluorescence staining results, we performed statistical analysis about relative fluorescence intensity (Fig 4A). These results showed that the numbers and intensity of positive signal in TGF-β1 overexpression group were fewest and minimal, however they were most in knockdown group. Additionally, we assessed the effects of TGF-β1 on the mRNA expressions of M1 marker (CD86) and M2 marker (Arg1) by RT-qPCR in plantar skin tissues. As RT-qPCR results, TGF-β1 decreased the expression of CD86 and increased the expression of Arg1, compared to negative control (Fig 4B-C). The data indicated that TGF-β1 reduced the infiltrated macrophages and promoted macrophage polarization in plantar skin tissues.

### TGF-β1 inhibited spinal microglia and astrocyte and signal pathway

**Fig 5:**
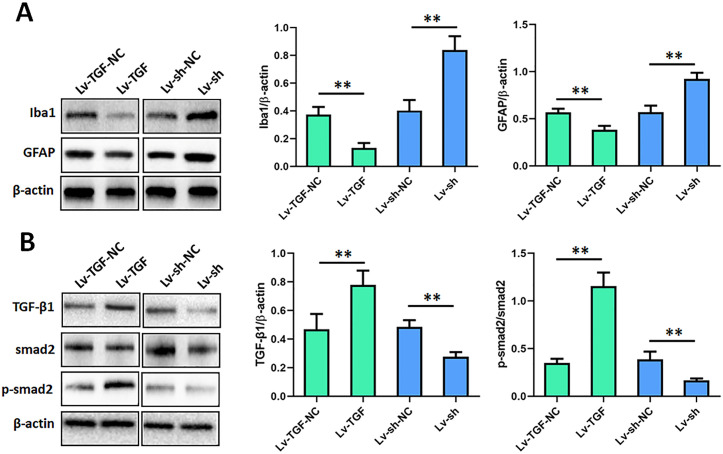
TGF-β1 inhibited spinal microglia and astrocyte and signal pathway. At 7 d after burn injury induced pain, Western blot was applied to detected the expressions of marker proteins of microglia and astrocyte, and TGF-β1 signal pathway. (A) the expressions of Iba1 and GFAP were detected by Western blot. (B) the expressions of TGF-β1, smad2 and p-smad2 were detected by Western blot. **p < 0.01. n = 3. Iba1, Ionized calcium binding adaptor molecule 1. GFAP, glial fibrillary acidic protein. TGF-β1, transforming growth factor-beta 1. shRNA, small hairpin RNA. Lv-sh, shRNA lentivirus. Lv-sh-NC, shRNA lentivirus negative control. Lv-TGF, TGF overexpression lentivirus. Lv-TGF-NC, TGF overexpression lentivirus negative control.

Microglia and astrocyte are involved in the regulation of inflammatory and neuropathic pain. To elucidate the effects of TGF-β1 on these glial cells, we assessed the effects of TGF-β1 on the expressions of Iba1 and GFAP, which are the marker proteins of microglia and astrocyte, by Western blot. Results showed that the expressions of Iba1 and GFAP were both remarkably dropped in TGF-β1 overexpression group (*p* < 0.01), compared to negative control group (Fig 5A). However, in TGF-β1 knockdown group, the oIba1 and GFAP expressions were both significantly raised. These data indicated that TGF-β1 inhibited spinal microglia and astrocyte.

We also detected the effects of TGF-β1 on the expressions of smad2 and p-smad2, which are the crucial factor in TGF-β1 signal pathway. The results revealed that TGF-β1 expression was prominently upregulated in Lv-TGF injection group, however its expression was sensibly downregulated in Lv-sh injection group. Moreover, the p-smad2/smad2 was dramatically increased in TGF-β1 overexpression group (*p* < 0.01), compared to negative control group (Fig 5B). And that the p-smad2/smad2 was significantly decreased in TGF-β1 knockdown group. These results indicated that TGF-β1 promoted the phosphorylation of smad2.

## Discussion

Pain is a ubiquitous symptom among burn patients, representing an inevitable consequence of the systemic pathophysiological changes induced by burn injury [5,37]. Thus, pain is one of the most common complaints of burn patients [5]. If not handled properly, pain can have a negative impact on burn treatment and wound healing [37,38]. Therefore, a better analgesic program is the basis of burn care, and it is urgent to discovery new analgesic therapy for burn induced pain. TGF-β1 is multifunctional cytokine. In the study, we expanded the multifunctional growth factor of TGF-β1 in burn injury pain, and found that TGF-β1 could relieve pain via alleviating inflammation.

During burn injury, excessive activation or increased excitability of primary sensory neurons leads to peripheral nerve sensibilization [39]. Previous studies on burn-induced pain primarily focused on the site of tissue damage. However, recent research has increasingly emphasized the role of peripheral nerve dorsal root ganglion (DRG) [40,41]. In addition to neurons, there are also a large number of glial cells in the nervous system. With the gradual deepening of research, a large number of reports have confirmed that non-neuronal cells (immune cells and glial cells) also play an important regulatory role in the occurrence and development of chronic pain [42,43]. Here, we investigated the effects of TGF-β1 on macrophages and glial cells by spinal intrathecal injection.

The process of burn generally includes tissue injury, inflammatory response, healing and remodeling [44]. Initially, burn causes protein denaturation and the destruction of plasma membrane integrity, which in turn leads to cell death and the release of cell contents, and finally promotes a severe inflammatory reaction [45]. This process will cause massive infiltration of inflammatory cells in peripheral tissues, and then produce a variety of inflammatory mediators [46]. On the one hand, inflammatory mediators cause the expansion of blood vessels at the injury site and the extravasation of tissue fluid, resulting in edema at the injured site; On the other hand, inflammatory mediators activate primary sensory neuron terminals located at the site of injury, leading to the release of neurotransmitters and pain [46,47].

IL-1β is mainly produced by macrophages and neutrophils, and can also be produced by primary sensory neurons [48]. Some studies have found that subcutaneous injection of IL-1β can induce significant thermal hyperalgesia and mechanical allodynia [49]. The content of IL-6 in burn tissue fluid was higher. Studies have shown that the upregulation of IL-6 at the burn site is related to inducing mechanical allodynia in rats [50]. Summer et al demonstrated that oligodeoxynucleotides antisense for gp130 can significantly attenuate burn induced thermal pain hyperalgesia, indicating that IL-6 played a crucial role in the regulation of burn induced pain [51]. In addition, IL-6 and its receptor both are expressed in primary sensory neurons. TNF-α is another inflammatory factor that exists in burn tissue fluid. The concentration level of TNF-α in the serum of burn patients is higher than that of health people. Under physiological conditions, TNF-α is mainly synthesized in mast cells. However, in the burn injured process, TNF-α can also be synthesized in a variety of cells, including keratinocytes, fibroblasts and monocytes [52]. Additionally, in burn induced peripheral nerve injury, TNF-α can also be synthesize in Schwann cells [53]. In the study, TGF-β1 could restrain pro-inflammatory factors (IL-1β, IL-6 and TNF-α) expressions and promote anti-inflammatory (IL-10) factors expressions in ipsilateral spinal cord. The data revealed that TGF-β1 relieved pain by regulating inflammatory factors.

Macrophages residing and circulating in tissues and organs play an important role in mammalian injury [54]. They help regulate inflammation and guide the inflammatory response toward repair and regeneration [55]. After tissue injury, macrophages can not only derive matrix metalloproteinases (MMP) to destroy the basement membrane to promote the movement of inflammatory cells to the site of tissue injury, but also produce chemokines to guide the initial recruitment of inflammatory cells [56]. Moreover, macrophages can phagocytose cell debris, invading organisms, neutrophils and other apoptotic cells after tissue injury. Macrophages involved in wound repair promote the production of a large number of growth factors, including platelet-derived growth factor (PDGF), insulin-like growth factor (IGF), and vascular endothelial growth factor (VEGF), which promote cell proliferation and vascular development [56]. And macrophages produce soluble mediators (such as TGF-β1) to stimulate local and recruited tissue fibroblasts to differentiate into myofibroblasts, promoting wound repair and the synthesis of extracellular matrix (ECM) components [57]. In the study, we found that burn caused the accumulation of macrophages at the injury site and TGF-β1 reduced the macrophages in plantar skin tissues.

A large amount of studies had confirmed that microglia participate in the regulation of inflammatory and neuropathic pain, and ultimately promote the formation of chronic pain [58]. There are two possible mechanisms for microgliosis: one is that the existing microglia proliferate in the spinal cord, because studies have shown that the expression of cell proliferation markers in microglia is up-regulated after nerve injury; Another mechanism is that injury will induce bone marrow-derived monocytes to infiltrate into the spinal cord and differentiate into microglia [58]. Astrocytes are the most abundantly glial cell type in the central nervous system, accounting for 20−40% of all glial cells. Astrocytes play an important role in the occurrence and development of chronic pain [59]. Peripheral nerve injury can induce the proliferation and hypertrophy of astrocytes in the spinal dorsal horn. Astrocytes are significantly activated in almost all chronic pain [59]. Studies have found that the expression of IL-1β in astrocytes was significantly up-regulated in inflammation and nerve injury models, while inhibiting the expression of IL-1β in spinal cord and brain could produce high-efficiency analgesic effect, and enhance the analgesic effect of morphine [59]. In the study, the expressions of Iba1 and GFAP were both significantly downregulated in TGF-β1 overexpression group. It indicated that TGF-β1 might inhibit spinal microglia and astrocyte to relieve pain.

In the study, only male mice were included. Albeit, there were many animal studies have shown that estrogens exert neuroprotective and neurogenesis effects *in vivo* and *in vitro* [60]. Future research could explore the application of TGF-β1 in female mice. The intrathecal space became an important venue for medical interventions in recent years, both in anesthesia and in many other medical specialties. In this study, spinal intrathecal injection with lentivirus was applied to overexpress TGF-β1. Although, the application of intrathecal injection is increasing. Rare and serious complications of undesired intrathecal injections have been reported, such as paraplegia [61]. Next, intravenous or local injection maybe taken into consideration. TGF-β1 recombinant protein also could be applied to inject.

To sum up, TGF-β1 alleviated burn injury induced pain by alleviating inflammatory response via TGF-β1/smad2 pathway. This study provides an experimental and theoretical basis for the potential clinical use of TGF-β1 as an anti-inflammatory and analgesic agent for burn injury.

## Supporting information

S1 File Uncropped images for all blot results. (PDF)
