# Supplementary figures and images for: TGF-β1 relieves burn injury induced pain by alleviating inflammation in mouse
Source: PLoS One. 2026 Feb 5;21(2):e0342029. doi: 10.1371/journal.pone.0342029 (PMC12875468; doi:10.1371/journal.pone.0342029)

Figure 5A

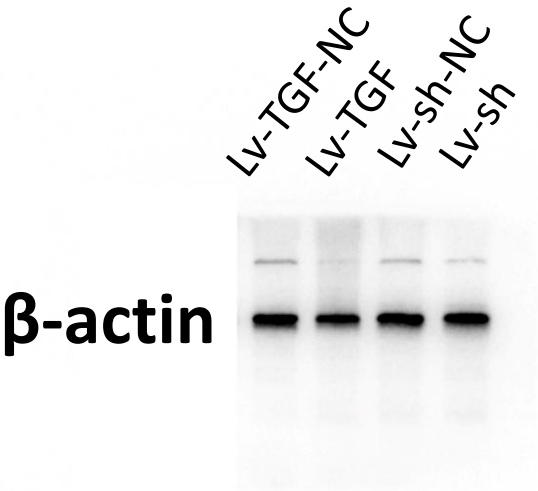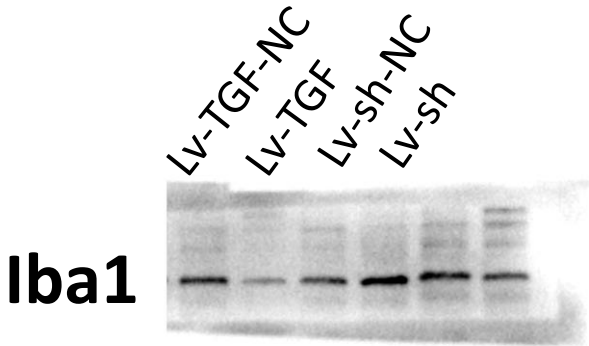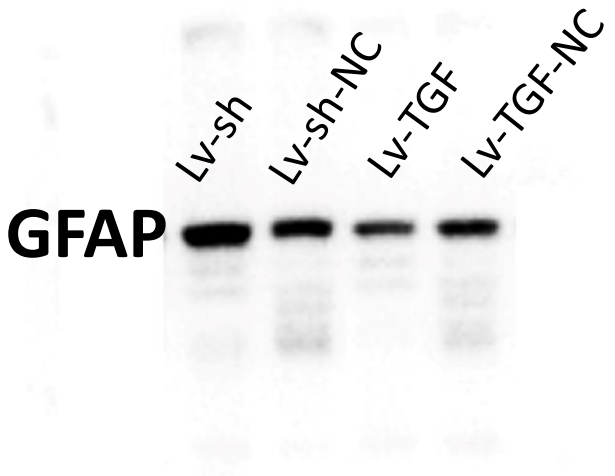

Figure 5B

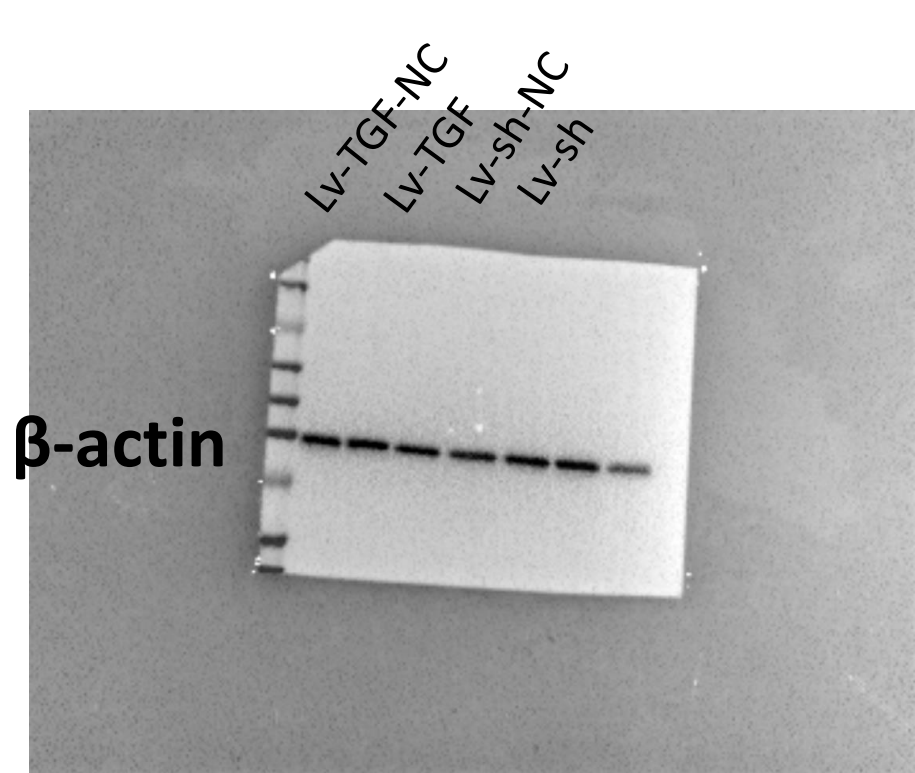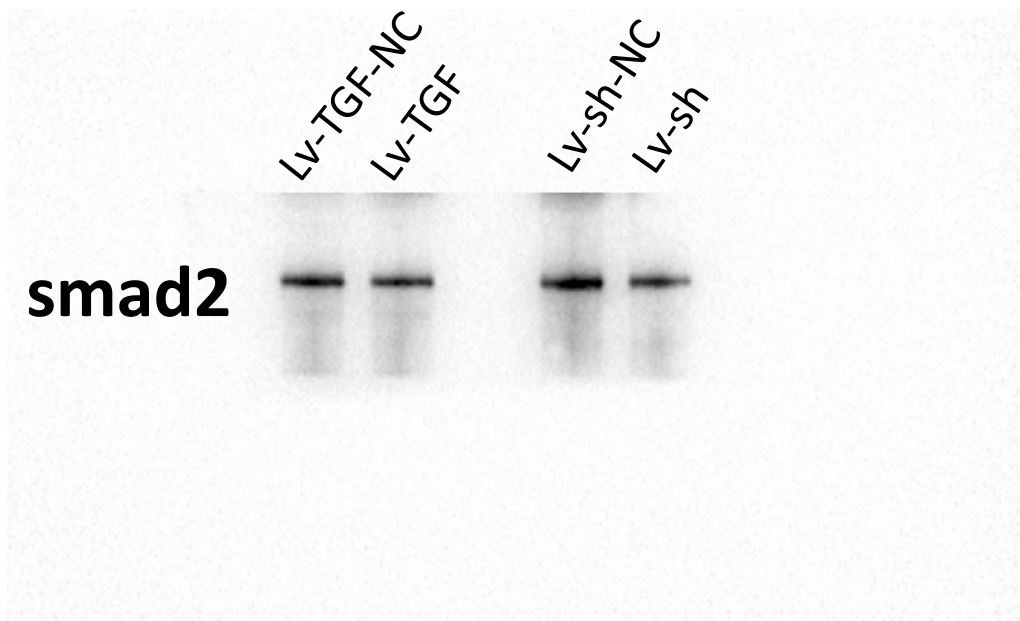

Figure 5B

p-smad2

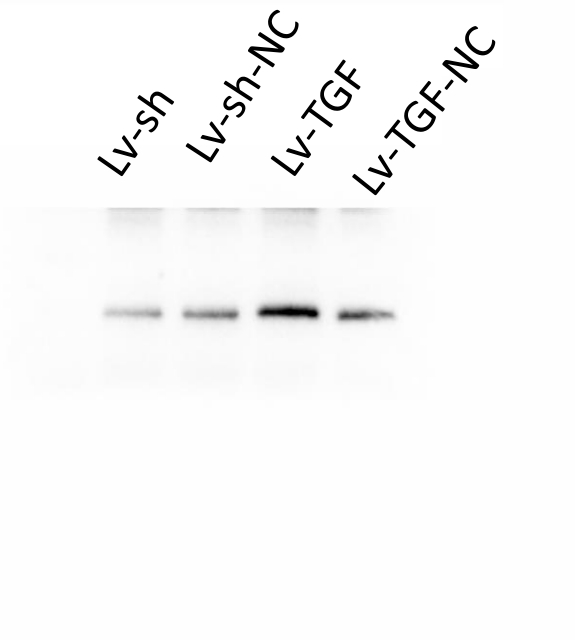

TGF-β1

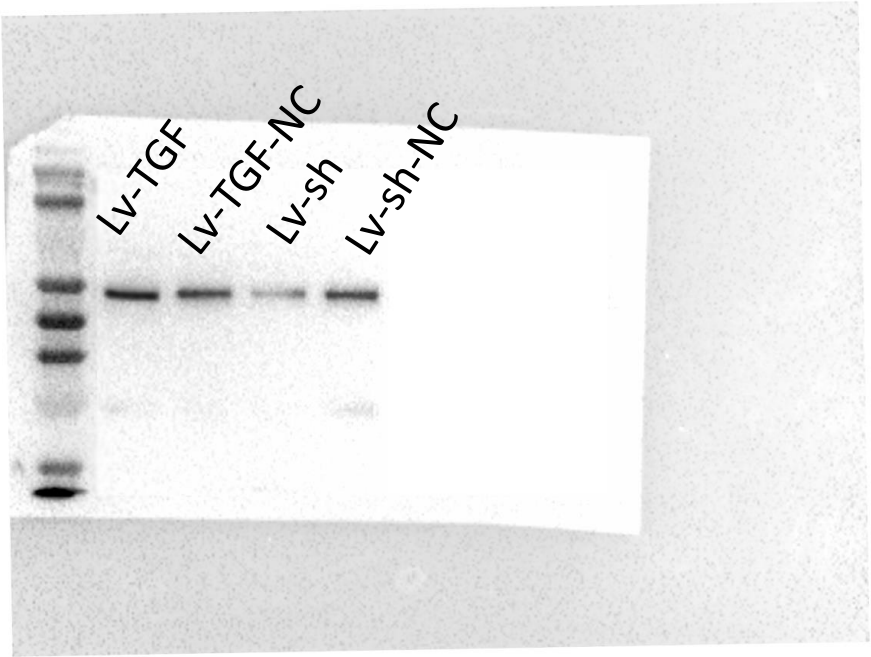

Supplement: S1 File — (PDF) [file pone.0342029.s001.pdf]
